# Smart-Data-Driven System for Alzheimer Disease Detection through Electroencephalographic Signals

**DOI:** 10.3390/bioengineering9040141

**Published:** 2022-03-28

**Authors:** Teresa Araújo, João Paulo Teixeira, Pedro Miguel Rodrigues

**Affiliations:** 1 CBQF—Centro de Biotecnologia e Química Fina—Laboratório Associado, Escola Superior de Biotecnologia, Universidade Católica Portuguesa, Rua de Diogo Botelho 1327, 4169-005 Porto, Portugal; teresa.mendonca.araujo@gmail.com; 2 CEDRI—Research Centre in Digitalization and Intelligent Robotics and UNIAG—Management Applied Research Unit, Instituto Politécnico de Bragança (IPB), Braganca, Campus de Sta Apolónia, Apartado 134, 5301-857 Bragança, Portugal; joaopt@ipb.pt

**Keywords:** Alzheimer disease, nonlinear multi-band analysis, electroencephalographic signals, classic machine learning, deep learning, wavelet packet, classification

## Abstract

Background: Alzheimer’s Disease (AD) stands out as one of the main causes of dementia worldwide and it represents around 65% of all dementia cases, affecting mainly elderly people. AD is composed of three evolutionary stages: Mild Cognitive Impairment (MCI), Mild and Moderate AD (ADM) and Advanced AD (ADA). It is crucial to create a tool for assisting AD diagnosis in its early stages with the aim of halting the disease progression. Methods: The main purpose of this study is to develop a system with the ability of differentiate each disease stage by means of Electroencephalographic Signals (EEG). Thereby, an EEG nonlinear multi-band analysis by Wavelet Packet was performed enabling to extract several features from each study group. Classic Machine Learning (ML) and Deep Learning (DL) methods have been used for data classification per EEG channel. Results: The maximum accuracies obtained were 78.9% (Healthy controls (C) vs. MCI), 81.0% (C vs. ADM), 84.2% (C vs. ADA), 88.9% (MCI vs. ADM), 93.8% (MCI vs. ADA), 77.8% (ADM vs. ADA) and 56.8% (All vs. All). Conclusions: The proposed method outperforms previous studies with the same database by 2% in binary comparison MCI vs. ADM and central and parietal brain regions revealed abnormal activity as AD progresses.

## 1. Introduction

There are many factors that have repercussions on ageing. During life, social and physical environment, as well as behavioural attitudes have a major impact on human ageing process. Nowadays, as a result of medical improvements, people are living longer—both an increase in the average life expectancy and a drop in the fertility rate has been observed. The World Health Organization (WHO) states that there are 125 million of people 80 years old or more, which is expected to increase to 434 million by 2050 [1].

Over the years, it is predictable that more ageing health problems will arise. Thus, it is crucial to consider not only the physical deterioration (the visible one), but also the nervous system deterioration. Concerning elderly people, it is proved that they have a greater propensity to develop neurodegenerative diseases such as dementia. Neurodegenerative disease is a serious clinical condition since it truly affects the nervous system functioning and progresses irreversibly. Epidemiological data state that 1 in 3 seniors dies with dementia [2,3].

Every 3 seconds, a new case of dementia is diagnosed. The WHO estimates that there are around 50 million people suffering from dementia worldwide. It is estimated that this value may reach about 82 million and 152 million in 2030 and 2050, respectively. In terms of geographic distribution, there is an expected higher rate of incidence in low- and middle-income countries compared with high-income countries [4].

Alzheimer Disease is known as the most common form of dementia, representing 65% of all dementia cases. It is characterised by the deterioration of human cognitive functions, affecting behaviour, language, memory and reasoning, among others. Alzheimer is a neurodegenerative disease in which the accumulation of certain toxic substances in the brain leads to the progressive death of neuronal cells. The major brain changes that AD causes are the accumulation of an atypical form of the tau protein within neurons, as well as the accumulation of the β-amyloid fragments outside neurons [5,6].

The AD progress can be described over four development stages: Pre-clinical, Mild Cognitive Impairment (MCI), Moderate AD and Severe AD. In the first stage, there are no associated symptoms (asymptomatic period). It is believed that AD arises at least 20 years before symptoms appear, hence the patient could stay in this initial phase for a long period of time. During the second phase, MCI, symptoms like memory lapses begins. The concept of MCI was created with the aim of including individuals with mild symptoms who might eventually progress to AD. It is stated that 6% to 25% of MCI patients later develop AD. The moderate stage is typically the longest one—frequent memory lapses and difficulty in performing daily routine tasks are faced, as well as other psychological symptoms. Lastly, the severe stage is the moment that patient loses the ability to interact with those who are around, having cognitive and motor functions extremely altered [5,7,8].

There are several factors that increase the risk of developing AD. Genetics, age, sex, lifestyle and other diseases are considered the main ones. Concerning family history, having one and two first-degree relatives with AD increases the risk of developing the disease by four and eight times, respectively. As it can be expected, dementia is scarce before the age of 65. Concerning sex, statistical data shows that AD is more common in women. As far as lifestyle habits are concerned, smokers and inactive people develop a propensity for this disease. The level of education also has impact since more years of education means a constant brain stimulation. There is also a strong evidence that having a healthy diet may reduce the probability of developing dementia. People diagnosed with depression and cardiovascular diseases are considered equally susceptible [5,9,10,11,12].

To date, there is no cure for this disease. However, there are several medical exams that support the diagnostic when someone reveals signs of dementia during life—Magnetic Resonance Imaging (MRI), Positron Emission Tomography (PET), Cerebrospinal Fluid (CFS) Analysis and Electroencephalogram (EEG). Besides that, Neurologists consider the medical history and perform several neurological and cognitive tests. It is important to note the true diagnosis of Alzheimer is only achieved with autopsy exam, after patient’s death [13].

EEG is a non-invasive and painless medical exam that aims to record and evaluate brain electrical activity. EEG signal is acquired by placing electrodes on the scalp and its frequency and amplitude values are contained in the range of 1 to 100 Hz and 10 to 100 µV, respectively. EEG enables to identify potential abnormalities in brain wave patterns [14]. Those abnormalities may be linked with brain illnesses such as dementia [15].

In the clinical branch, it is possible to evaluate brain abnormalities in a patient’s brain activity by analysing the EEG signal frequency. In this way, it is considered five conventional frequency bands—delta, theta, alpha, beta and gamma. Delta waves (δ) are the slowest and highest amplitude waves, belonging to the frequency range of 0.5 to 4 Hz. These waves are related to events such as deep sleep and wakefulness. Theta waves (θ) have an amplitude greater than 20 µV and are contained within the frequency range of 4 to 8 Hz. The presence of these waves is associated with moments of emotional stress, deep meditation and creative inspiration. Alpha waves (α) belong to the frequency and amplitude range of 8 to 13 Hz and 30 to 50 µV, respectively. When a person is with the eyes closed or in a relaxed state, these waves appear mostly in the occipital lobe. Beta waves (β) refer to rhythms in the frequency range of 13 to 30 Hz, having amplitude values less than 30 µV. This wave type is commonly observed in the brain of a healthy adult and it is associated with active brain activity, active thinking and problem solving. Finally, gamma waves (γ) are the fastest and lowest amplitude waves, lying above the frequency of 30 Hz (normally the upper limit is 100 Hz). The activity of these waves are of rare occurrence—a brain disease can be confirmed by an abnormal presence of this rhythm [16,17].

An AD patient reveals changes in the EEG signal compared to the normal patterns observed in a healthy individual. The main phenomenon is named slowing effect. As the disease progresses, there is an increase in power in the lower frequency bands (δ and θ), as well as a decrease in power in the higher frequency bands (α, β and γ). Additionally, shift-to-the-left phenomenon is also observed. A shift of the power spectrum peak at the alpha band to the lower frequency bands occurs. The surface distribution of these rhythms becomes emphatic in anterior regions, instead of in the occipital region (healthy subject). Briefly, these signal power modifications are essentially due to the lack of acetylcholine in the AD brain. Insufficient amounts of acetylcholine result in failures in the synchronisation of synaptic potentials [8,18,19].

Given the large number of people affected and the inherent severity, it is urgent to find a method capable of assisting in the AD early stages diagnosis. For this reason, the main goal of the present work is to create an intelligent system that can be useful for that purpose—a tool that could detect anomalies in the EEG signal of an Alzheimer carrier during the asymptomatic period so that the disease evolution can be delayed. This paper proposes a new method based on a nonlinear multiband analysis of the EEG signals. By the extraction of relevant features, it was feasible to perform the data classification per electrode by means of Classic ML and DL.

In terms of structure, this paper is organized in five main sections: In Section 2, the EEG database is described. Thereafter, the methodology concerning the signal processing and the classification procedure is explained in Section 3. The obtained results and the inherent discussion were covered in Section 4. Lastly, Section 5 makes remarks about conclusions.

## 2. Materials

**Figure 1:**
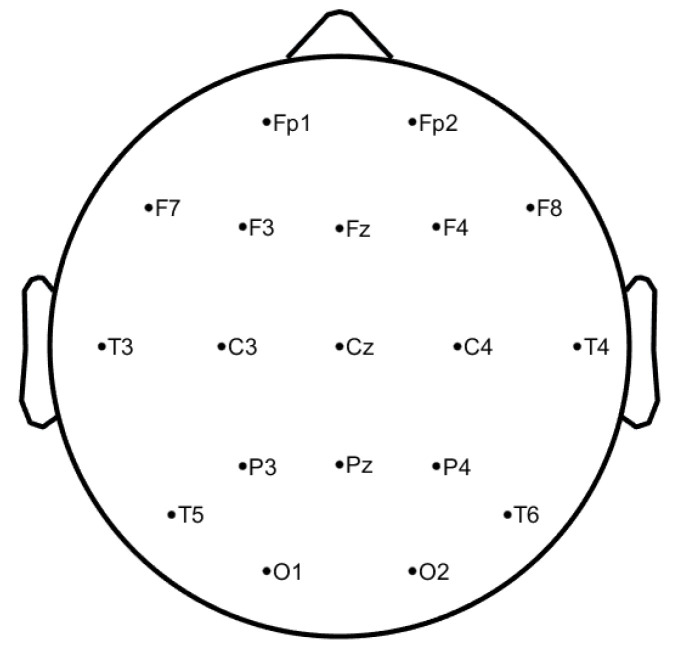
10–20 EEG system—electrodes positions at scalp level.

The EEG signals have been collected at Hospital de São João in Porto, Portugal, with the approval of the local ethics committee and the hospital’s administration board within the project CES198-14. The data were acquired at a sampling rate of 256 Hz through 19 electrodes placed on the scalp, using the common reference electrode at CPz, according to the 10–20 system (see Figure 1, image generated by authors in Matlab using EEGLab [20]), covering the frontal (Fz, FP1, FP2, F3, F7), temporal (T7, T8), parietal (Pz, P3, P4, P7, P8, PO7, PO8), occipital (O1, O2), and central (Cz, C3, C4) regions, resulting in 19 waveforms per exam. During the acquisition, it was ensured that all the study subjects were relaxed and with the eyes closed. The DC component was removed. It is important to note that only signals without any kind of artefacts are selected.

This database includes 38 subjects split into 4 distinct groups—11 healthy subjects called healthy controls (C), 8 MCI, 11 AD mild and moderate (ADM) patients and 8 AD advanced (ADA) patients. The average Mini Mental State Examination (MMSE) and age average of each group are presented in Table 1. MMSE is the most common exam used in clinics and hospitals to assess patients’ major cognitive domains, such as visuospatial abilities, attention, memory and language. Its scores range from 0 to 30. Scores on the higher end indicate a higher cognitive function, while lower scores mean more severe cases of dementia [21].

## 3. Methods

**Figure 2:**
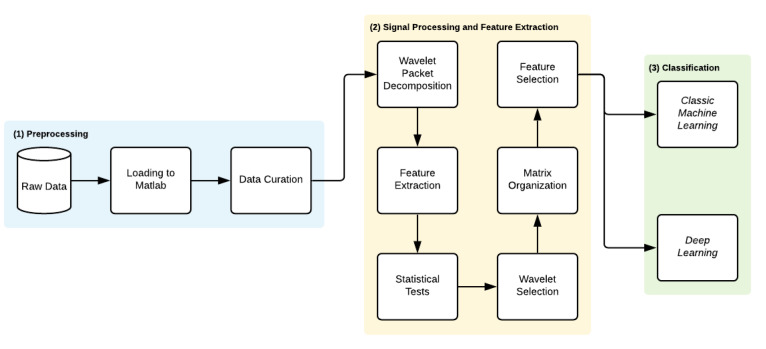
Methodology workflow.

The proposed methodology is divided into three main steps—(1) Preprocessing, (2) Signal Processing and Feature Extraction and (3) Classification. Figure 2 summarizes the methodology implementation steps.

### 3.1. Preprocessing

The EEG signals belonging to the database were loaded into the Matlab software and then split in 5 seconds segments of 1280 samples per segment. Each signal has 19 channels and the number of segments is variable between subjects. Thereafter, each segment was normalized per channel according to Equation 1. It is important to emphasize that these signals had already been submitted for noise removal and they were digitally filtered by a 1–40 Hz band-pass filter.

(1) $$y\left(n\right)=\frac{x(n)}{\sum_{n=0}^{N-1}x^{2}\left(n\right)}$$

### 3.2. Signal Processing and Feature Extraction

This section describes the set of features extracted from each segment of the EEG signal.

#### 3.2.1. Wavelet Packet Decomposition

Wavelet analysis is a method that benefits from the power of a variable-sized window, being able to analyse a specified area of a large signal. On the one hand, the use of a large window size (long time intervals) provides the capture of low-frequency information. On the other hand, the capture of high-frequency information is achieved by applying a reduced size window (short time intervals) [22,23].

This technique aims to decompose the original waveform (mother wavelet) into several versions of it (single wavelet). Basically, the signal is decomposed into low (approximation coefficients) and high frequencies (detail coefficients). Generally, the low frequency components are the most important since they characterize the signal identity. In turn, the high frequency components analysis reflects the more specific details of a signal [23].

More specifically, Discrete Wavelet Packet Transform (DWPT) provides a simultaneous decomposition of low and high frequencies. Unlike the Discrete Wavelet Transform (DWT), it also considers high frequencies after the 1st level of decomposition. This procedure is iterative, as the signal is successively filtered both by a low-pass and a high-pass filter (obtaining approximation and detail coefficients, respectively) [24,25].

**Figure 3:**
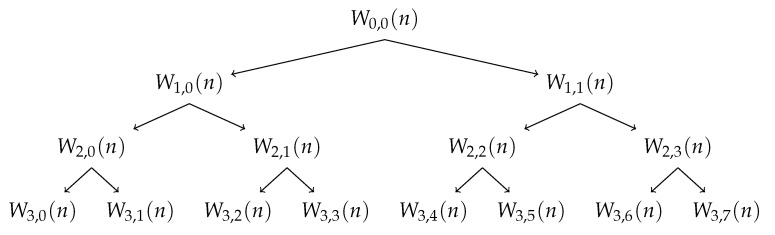
Wavelet Packet Tree - Example of decomposition until scale level 3.

The *DWPT* equation is defined as follows:

(2) $$W_{s,m}\left(n\right)=\left\{\begin{array}{cc} \sqrt{2^{s}}\sum_{k=0}^{N-1}h_{0}\left(2n-k\right)W_{\left(s-1\right),\left({}^{m}\;{/}_{2}\right)}\left(k\right), & \mathrm{if}\;m\;\mathrm{even}\\ \sqrt{2^{s}}\sum_{k=0}^{N-1}h_{1}\left(2n-k\right)W_{\left(s-1\right),\left({}^{m-1}\;{/}_{2}\right)}\left(k\right), & \mathrm{if}\;m\;\mathrm{odd} \end{array}\right.$$

where *s* is the scale of decomposition ($s=\{0,1,2,...,log_{2}\left(n\right)\}\in Z$), *N* corresponds to the signals length, *m* denotes the position in the tree at scale *s* ($m=\{0,1,2,...,2^{j}-1\}$), *n* are the samples (n={0,1,2,...,N−1}), and *k* represents the time (k∈R). $W_{s,m}$ are the coefficients of the Wavelet Packet at the scale *s* and position *m* as can be seen in Figure 3. $h_{0}$ and $h_{1}$ are the high-pass and low-pass Wavelet filters, respectively. These filters depend from the selected wavelet [24]. It must be pointed out that $W_{0,0}$ is the original normalized signal — y(n), the sub-band signal $W_{s,m}\left(n\right)$ has its frequency limited in $\left[m\pi /2^{s},\left(m+1\right)\pi /2^{s})\right]$, where π is the normalized angular frequency. The filters applied vary according to the mother wavelet family and subfamily [24].

In this work, the DWPT is applied until scale level 6 to each 5 s EEG segment per channel for computing 18 nodes through Wavelet Packet Tree, i.e., 18 EEG frequency subbands have been reached, analysing on this way all frequency band range that includes the conventional bands δ, θ, α, β and γ.

#### 3.2.2. Non-Linear Analysis

According to the nonlinear dynamic theory, a complex system (such as the human brain) is characterized by nonlinear dynamic properties. Due to physiological events, the brain environment is constantly changing over time. Consequently, brain waves exhibit a nonlinear and chaotic behavior. Additionally the degree of complexity of the brain represents the time series randomness. Depending on the intensity of the brain activity, the EEG signal can be more or less complex, containing more or less information per signal fragment. Briefly, many methods of extracting nonlinear features have been increasingly explored, making them a powerful approach for EEG signal characterization. Indeed, through the detection of patterns in the time series, it is possible to infer about EEG signal behavior and predict the same kind of occurrences in the future [26].

The present method suggested the extraction of 20 nonlinear and statistic features from each node and from each one of the 19 channels of all participants’ signal segments with the purpose of accomplish a non linear and multiband analysis. Generally, the non-linearity characteristics correspond to entropy, exponents and fractal dimensions parameters.

##### Energy

The sub-bands energies are the most used EEG features for detecting AD due to the power shift effect from high to low frequencies that progressively occur as AD progresses. The energy of a signal x(n) is defined as

(3) $$\begin{array}{c} EN=\sum_{n=1}^{N}{\left|x\left(n\right)\right|}^{2} \end{array}$$

##### Entropy

Entropy concept arises with the intention of describing the molecules distribution in a given system. This thermodynamic definition explains how molecules are organized, considering the size and atomic configuration of each one. Thus, the assessment of entropy allows the quantitative evaluation of the degree of randomness and uncertainty of a given sequence of data. Entropy is a measure that considers the amount of energy present in the complex system. In fact, entropy features are commonly used, as their analysis allows us to accurately evaluate the non-linear behaviour characteristic of the EEG signals [17,24,26]. Several measures of entropy were used (Shannon, logarithmic, norm, approximate, sample, and permutation entropy), leaving the feature selections process elect the one(s) more relevant for the EEG classification.

- Shannon Entropy [27]:
  (4) $$\begin{array}{c} ShE=-\sum_{n=1}^{N}{\left|x\left(n\right)\right|}^{2}{log[|x\left(n\right)|}^{2}] \end{array}$$
- Logarithmic Entropy [28]:
  (5) $$\begin{array}{c} LE=\sum_{n=1}^{N}{log[|x\left(n\right)|}^{2}] \end{array}$$
- Norm Entropy:
  (6) $$\begin{array}{c} NE=\sum_{n=1}^{N}{\left|x\left(n\right)\right|}^{l} \end{array}$$
  where *l* represents the normalized power equal to 1.1 on present study [29].
- Approximate Entropy:
  (7) $$\begin{array}{c} AE\left(e,r,N\right)=H^{e}\left(r\right)-H^{e+1}\left(r\right) \end{array}$$
  where *N* is the data length (suggested to be 1000 of the signal standard deviation), *r* is the similar tolerance (between 0.1 and 0.25) and *e* represents the embedding dimension (between 2 and 3). *H* is the Heaviside function that results from intermediate calculations [29].
- Sample Entropy:
  (8) $$\begin{array}{c} SE\left(e,r,N\right)=-\ln\frac{A^{e+1}\left(r\right)}{B^{e}\left(r\right)} \end{array}$$
  where,
  (9) $$\begin{array}{c} A^{e+1}\left(r\right)=d\left[x_{e+1}\left(i\right),x_{e+1}\left(j\right)\right]<r; \end{array}$$
  and
  (10) $$\begin{array}{c} B^{e}\left(r\right)=d\left[x_{e}\left(i\right),x_{e}\left(j\right)\right]<r; \end{array}$$
  as the factors *N*, *r*, *e* are the same used in Approximate Entropy, the recommended values are similar, and *d* is the Chebyshev distance between two sets of simultaneous data points [29].
- Permutation Entropy:
  (11) $$\begin{array}{c} PE\left(e\right)=-\sum_{j=1}^{m!}p_{j}\ln p_{j} \end{array}$$
  where *e* is the embedding dimension and $p_{j}$ represents the probability of the $j^{th}$ permutation occurring [30].

##### Chaos Theory

Chaos theory is an approach closely related to dynamic systems. A dynamic system does not share the properties of a system in equilibrium, wherefore certain unpredictable disturbances may influence its behavior. In this way, those perturbations cause the system transition from one state to another. The concept of phase space represents the set of all possible states through which a dynamic system can pass over time. There are two main exponents which provide a comprehensive framework of chaos [17,31].

- Hurst Exponent:
  (12) $$\begin{array}{c} HE=\frac{\epsilon -1}{2} \end{array}$$
  where ϵ derives from power-law [26].
- Lyapunov Exponent:
  (13) $$\begin{array}{c} LE\left(x_{0}\right)=\lim_{n\to \infty }\frac{1}{n}\sum_{k=1}^{n}\ln\left|f^{\prime }\left(x_{k}-1\right)\right| \end{array}$$
  where $f^{\prime }$ is the derivative of the iterator function *f* [17].

##### Fractal Analysis

Fractal structures present self-similarity properties that enable detailing both irregular processes and structures. Regarding brain activity study, fractal geometry has been showing to be a useful approach to identify neurological pathologies. In fact, monitoring the self-similarity of the brain rhythms allows the characterization of the patient clinical condition. Fractal feature extraction such as the Higuchi fractal dimension is considered to be helpful in EEG signal classification [31].

- Higuchi Exponent:
  (14) $$\begin{array}{c} L\left(k\right)\alpha k^{-D} \end{array}$$
  where *k* indicates the time interval, L(k) is the length of the curve in the *k* time interval and *D* is the Higuchi Exponent [32].

#### 3.2.3. Feature Extraction Process

For each 38 study participants, 10 non-linear features (Energy, Shannon Entropy, Logarithmic Entropy, Approximate Entropy, Sample Entropy, Permutation Entropy, Norm entropy, Hurst Exponent, Lyapunov Exponent, and Higuchi Exponent) are calculated from the 18 DWP nodes signals of all 5 s segments per channel. At the same time, 10 time-series statistics used for a fast signal analysis, such as Maximum, Mean, Median, Mode, Kurtosis, Standard Deviation, Variance, Skewness, Root Mean Square and Asymmetry [33], have been also extracted from each 18 DWP nodes. Thus, a total of 20 features have been computed in each DWP node 5 s signal analysis per channel.

Treating the 20 extracted features of all segments per DWT Node analysis as time series distributions, 3 statistics have been used to compress them along the channel, reducing in this way the dimensionality of the problem. Mean, variance (var) and standard deviation (sd) have been picked for this purpose.

At the end of the process, each DWT node analysis per channel produced 60 features (20 time series features digitally compressed by 3 statistics, respectively).

### 3.3. Wavelet Selection Process

**Figure 4:**
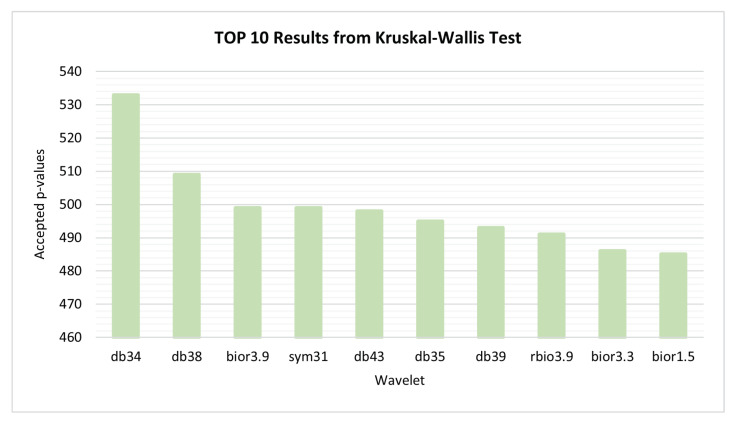
Kruskal–Wallis test—10 best performances achieved by wavelets.

Since the values of each feature depend on the wavelet used in the signal decomposition, a search to find the wavelet that results in features with greater discriminant capacity for all comparisons at the same time was performed. The wavelet families evaluated have been Biorthogonals, Reverse Biorthogonals, Daubechies, Coiflets, Symlets and Fejer-Korovkin. The values of each feature computed from the segments have been organized for each combination of study groups pair, wavelet, feature, statistic and node. Within each combination, the values are normalized using z-score [34]. Then, for each combination, the normalized values are applied to the the Kruskal–Wallis test. Figure 4, presents the number of features with accepted p-value for each mother wavelet. The wavelet which optimizes and emphasizes the Alzheimer’s activity along its progresses was the db34, thus this wavelet has been chosen for feature selection and classification procedure steps.

### 3.4. Feature Selection and Classification Procedure

The main goal of this study is to infer about the capacity of Classic ML and DL methods to evaluate the scalp activity evolution of AD along its different stages. For the propose, a set of Classic ML tools, such as decision trees (DT), support vector machines (SVM), Naive Bayes, k-nearest neighbors (KNN), logistic regression and discriminant analysis and one Deep Learning classifier (Convolution Neural Networks, CNN) have been used (please check Table 2 for more details).

In each group comparison, to find the best feature combination for classifiers entries the non-normalized values of each feature, computed from all segments of all channels of all research participants, were splited by each study group pair per channel.

In each comparison, this results in 1080 features values (60 features per DWT node analysis × 18 DWT nodes) per each subject channel analysis. Within each comparison, the values are normalized by z-score [34]. After being normalized, the features extracted are applied to a f-score algorithm [35] in order to select the best set of features per comparison according with the maximum accuracy achieved on the 19 channels analysis. Thus, 11 different features combinations were applied to the entries of Classic ML and DP algorithms: 2 features, 3 features, 4 features, 5 features, 10 features, 15 features, 20 features, 5% of features, 10% of features, 20% of features and 100% of features. In all cases, in order to verify the generalization capacity of the classifiers, a leave-one-out cross-validation procedure is used. Due to the dataset limited size, all data have been used in cross-validation procedure, using the leave-one-out methodology. Table 3 presents the maximum accuracy achieved on the 19 classifications per channels obtained for each binary comparison. It was concluded that the range up to 5 features contains the highest discriminative power in all comparisons.

## 4. Results and Discussion

The final results of the classification for each comparison case are shown in Table 4, where the best classifier, the maximum accuracy and the electrode scalp position are indicated per classifier modality (Classic ML and Deep Learning).

Analysing the results presented Table 4, the binary classification of C vs. MCI exhibits a maximum accuracy of 78.9% on P7 and Pz electrodes, being noticeable that those are the ones with the greatest discriminate power. Regarding this comparison, the classifier which presented this best performance was Decision Tree (Fine, Medium and Coarse Tree). In turn, DL selected the P8 electrode as the one which present more significant differences between groups, reaching 78.9% as maximum accuracy.

Regarding Classic ML, and when comparing C vs. ADM, it is observed that the C4 and P7 electrodes present a maximum accuracy of 81.0%, being the ones that reveal more significant differences. The classifiers Cubic SVM and Fine Gaussian SVM were the ones who correspond to the highest accuracy result. In contrast, through DL, a maximum accuracy of 76.2% was achieved in the Pz electrode, this being the one that corresponds to the scalp region with the highest discriminative power between subjects.

Concerning Classic ML, the binary classification of C vs. ADA exhibits a maximum accuracy of 84.2% visible in the F7, C4 and T8 electrodes, being noticeable that those are the ones with the greatest discriminative power. Regarding this comparison, the classifiers which presented this best performance were Linear SVM and Gaussian Naive Bayes. In turn, DL selected the F7 and F8 electrodes as the scalp regions which present more significant differences between groups, reaching 78.9% as maximum accuracy.

Regarding Classic ML, and when comparing MCI vs. ADM, it is observed that the P7 electrode present a maximum accuracy of 88.9%, being the one that reveals more significant differences. The classifier Cosine KNN was the one who correspond to the highest accuracy result. In contrast, through DL, a maximum accuracy of 83.3% was achieved in the Pz electrode, this being the one that corresponds to the scalp region with the highest discriminative power between subjects.

Concerning Classic ML, the binary classification of MCI vs. ADA exhibits a maximum accuracy of 93.8% visible in the O1 electrode; it is noticeable that this is the one with the greatest discriminative power. Regarding this comparison, the classifier which presented this best performance was Decision Tree (Fine, Medium and Coarse Tree). In turn, DL selected the P4 electrode as the scalp region which present more significant differences between groups, reaching 93.8% as maximum accuracy.

Regarding Classic ML, and when comparing ADM vs. ADA, it is observed that the F3, F8, C3, C4, O1 electrodes present a maximum accuracy of 77.8%, being the ones that reveal more significant differences. The classifiers Ensemble Subspace Discriminant and Fine KNN were the ones who correspond to the highest accuracy result. In contrast, through DL, a maximum accuracy of 72.2% was achieved in the Fz, F4 and C4 electrodes, these being the ones that correspond to the scalp regions with the highest discriminative power between subjects.

Concerning Classic ML, the multiclass classification of All vs. All exhibits a maximum accuracy of 56.8% visible in the Pz electrode, being noticeable that this is the one with the greatest discriminative power. Regarding this comparison, the classifier which presented this best performance was Medium Gaussian SVM. In turn, DL selected the Pz electrode as the scalp region which present more significant differences between groups, reaching 51.4% as the maximum accuracy.

**Figure 5:**
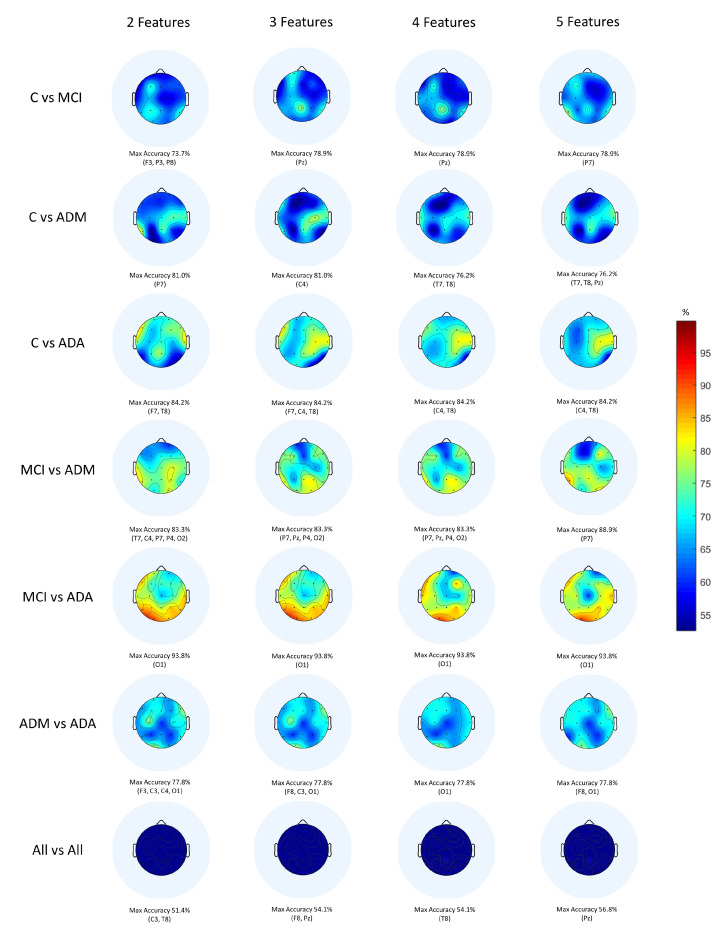
Topographic maps provided by a Classic ML classification.

In fact, Classic ML shows better results than DL with the exception of 2 out of 7 comparisons. This being the best technique, it is relevant to deepen the results obtained. To this end, the topographic maps were elaborated (Figure 5) with the Classic ML results in order to visualise the scalp regions that present the best values of accuracy.

When observing the topographic maps, it is possible to conclude that C4 and P7 are the electrodes which best distinguish the differences between subjects. Those electrodes correspond to the brain central region and to the parietal lobe, respectively. Depending on the disease stage and which stages are being compared, the area which is considered to be the most affected may vary. Although the temporal lobe is typically responsible for memory functions, it does not mean that the other scalp regions are not equally affected by the disease. According to Siuly and Zhang [16], the parietal area is very important in recognition and orientation. Actually, AD patients tend to lose these abilities, for example, at the moments when they do not recognise their family or when they feel lost in a physical space they previously knew. This conclusion sustains the results obtained.

Additionally, it is crucial to reflect on the work considered closest to this one in order to draw some further conclusions. To this end, Table 5 and Table 6 present a direct comparison between the developed work and others—same EEG database and different EEG database:

1 Compared to other methods of diagnosing AD through EEG signals from the same database (Table 5), the proposed method outperformed the study developed by Rodrigues et al. [19] by 2% in the binary comparison MCI vs. ADM. It can be seen that CNNs have never been applied to this dataset, so this work is the first and the only one that follows this approach. Indeed, this works presents added value to the scientific community, as it has the potential to be improved and become a powerful tool for AD diagnosis in all its stages.
2 Compared to other techniques of diagnosing AD through EEG signals from different databases (Table 6), it is observed that the present study outperformed the work carried out by Fiscon et al. [36] by 13% in the pair MCI vs. AD. It is noteworthy that the present study has the peculiarity of being the only one that applied the F-score technique, so it may have highly contributed to the good classification results.

In general, Classic ML proved to have more capacity to identify AD activity along its progression than DL. One of the reasons for this happening is that DL is commonly used for analysing large amounts of data. According to Esteva et al. [38], and particularly regarding the medical field, DL methods are benefic because of the capacity to generate sheer volume of data. In fact, this database only contains 38 participants, so the simpler methods (such as Classic ML) were sufficient to get better results than CNN.

## 5. Conclusions

AD is a neurodegenerative disease marked by a rapid progression, leading to total loss of cognitive functions. As with other types of dementia, the prevalence is increasing and forecasts show no improvements. During the early stages, patients do not have symptoms—the disease is silent. As soon as the symptomatic phase begins, there is a worsening of the clinical condition. Since AD is considered one of the most severe diseases and there is still no cure, the main goal of this work was to develop a strong system proficient of assisting in the AD diagnosis.

Hence, and taking benefit from Wavelet Packet Transform for multi-band analysis, several statistics and nonlinear features were extracted from EEG signals. The most interesting features were used to evaluate the significant differences between the study groups through Classic ML and DL classification methods. The main results indicate that Classic ML algorithms showed a higher discriminant power to emphasize AD activity than DL, except in the binary comparisons C vs. MCI and MCI vs. ADA where they achieved exactly the same accuracy.

In conclusion, the aim of this work was accomplished according to what was expected, since it corroborated the state of the art, having exceeded some discriminant accuracies comparing to others studies. Regarding the state of the art with the same EEG database, the proposed method outperforms previous works by 2%, in the binary comparison MCI vs. ADM. It is relevant to highlight that the remaining comparisons performed (C vs. MCI, C vs. ADM, C vs. ADA, MCI vs. ADA, ADM vs. ADA and All vs. All) did not show better results. Indeed, this improvement reflects the impact that this tool can have, particularly in distinguishing these two consecutive stages of AD.

## Figures and Tables

**Table 1:** EEG Database.

Subjects	C	MCI	ADM	ADA
#	11	8	11	8
Age Average	74	80	79	79
MMSE Average	28.68	26.29	18.89	11.50

**Table 2:** Used classifiers and optimal parameters.

Classifier		Optimal Parameters
Decision Trees	Fine Tree—FT	Maximum number of splits = 150
	Medium Tree—MT	Maximum number of splits = 150
	Coarse Tree—CT	Maximum number of splits = 150
Discriminant Analysis	Linear Discriminant—LD	Covariance structure: Full
	Quadratic Discriminant—QD	Covariance structure: Full
	Logistic Regression - LR	Covariance structure: Full
Naive Bayes	Gaussian Naive Bayes— GNB	-
	Kernel Naive Bayes—KNB	-
SVM	Linear SVM—LSVM	Box constraint level = 3
	Quadratic SVM—QSVM	Box constraint level = 3
	Cubic SVM—CSVM	Box constraint level = 4
	Fine Gaussian SVM—FGSVM	Box constraint level = 3
	Medium Gaussian SVM—MGSVM	Box constraint level = 3
	Coarse Gaussian SVM—CGSVM	Box constraint level = 1
KNN	Fine KNN—FKNN	Number of neighbors = 3
	Medium KNN—MKNN	Number of neighbors = 3
	Coarse KNN—CKNN	Number of neighbors = 3
	Cosine KNN CosKNN	Number of neighbors = 3
	Cubic KNN—CubKNN	Number of neighbors = 3
	Weighted KNN—WKNN	Number of neighbors = 3
Ensemble	Boosted Trees—BossT	Maximum number of splits = 150
	Bagged Trees—Bagt	Maximum number of splits = 150
	Subspace Discriminant—SubD	Covariance structure: Full
	Subspace KNN—SubKNN	Number of neighbors = 3
	RUSBoosted Trees—RUSBT	Maximum number of splits = 150
CNN		imageInputLayer = 1
		convolution2dLayer = 1
		reluLayer = 1
		fullyConnectedLayer = 3
		softmaxLayer = 1
		classificationLayer = 1
		Training algorithm = adam
		Max epochs = 1000

**Table 3:** Features combination.

Features	Maximum Accuracy					
	C-MCI	C-ADM	C-ADA	MCI-ADM	MCI-ADA	ADM-ADA
1080	73.7%	76.2%	68.4%	83.3%	87.5%	72.2%
20%	73.7%	76.2%	78.9%	88.9%	93.8%	72.2%
10%	78.9%	76.2%	78.9%	88.9%	93.8%	72.2%
5%	78.9%	76.2%	78.9%	83.3%	93.8%	77.8%
20	73.7%	81.0%	78.9%	88.9%	93.8%	77.8%
15	78.9%	81.0%	78.9%	88.9%	93.8%	77.8%
10	78.9%	76.2%	78.9%	88.9%	87.5%	77.8%
5	78.9%	76.2%	84.2%	88.9%	93.8%	77.8%
4	78.9%	76.2%	84.2%	83.3%	93.8%	77.8%
3	78.9%	81.0%	84.2%	83.3%	93.8%	77.8%
2	73.7%	81.0%	84.2%	83.3%	93.8%	77.8%

**Table 4:** Classic Machine learning vs. Deep Learning classification.

Comparison	Classic ML	Accuracy (Position)	DL	Accuracy (Position)
C vs. MCI	FT, MT, & CT	78.9% (P7 & Pz)	CNN	78.9% (P8)
C vs. ADM	CSVM & FGSVM	81.0% (C4 & P7)	CNN	76.2% (Pz)
C vs. ADA	LSVM & GNB	84.2% (F7, C4 & T8)	CNN	78.9% (F7 & F8)
MCI vs. ADM	CosKNN	88.9% (P7)	CNN	83.2% (Pz)
MCI vs. ADA	FT, MT & CT	93.8% (O1)	CNN	93.8 (P4)
ADM vs. ADA	FKNN & SubD	77.8% (F3, F8, C3, C4 & O1)	CNN	72.2% (Fz, F4 & C4)
All vs. All	MGSVM	56.8% (Pz)	CNN	51.4% (Pz)

**Table 5:** Comparison with previous works with the same EEG database.

Study	Signal Processing	Features	Feature Selection	Best Classifier	Classification Accuracy
[18]	Multiband Spectral Analysis via DWT	RP, Spectral Ratios, Maxima, Minima and Zero Crossing	KW Test	ANN	C vs. MCI—77%
					C vs. AD—95%
					MCI vs. AD—83%
					All vs. All—90%
[19]	Multiband Cepstral and Lacstral Analysis via DWT	Cepstral and Lacstral Distances	Genetic Algorithms	ANN	C vs. MCI—98%
					C vs. ADM—96%
					C vs. ADA—96%
					C vs. ADM-ADA—96%
					MCI vs. ADM—87%
					MCI vs. ADA—99%
					MCI vs. ADM-ADA—94%
					All vs. All—96%
Present Study	Nonlinear and Multiband Analysis via DWPT	Nonlinear and Statistic Parameters	F-score	SVM	C vs. MCI—79%
					C vs. ADM—81%
					C vs. ADA—84%
					MCI vs. ADM—89%
					MCI vs. ADA—94%
					ADM vs. ADA—78%
					All vs. All—57%

**Table 6:** Comparison with previous works with different EEG databases.

Study	Signal Processing	Features	Feature Selection	Best Classifier	Classification Accuracy
[36]	Fourier and Wavelet Analysis via FFT and DWT	Fourier and Wavelet Coefficients	Not applied	DT	C vs. AD—83%
					C vs. MCI—92%
					MCI vs. AD—79%
[37]	Multiband Analysis via DWT and EMD	Variance, Kurtosis, Skewness, Shannon Entropy, Sure Entropy and Hjorth Parameters	Not applied	KNN	C vs. AD1 vs. AD2—98%
Present Study	Nonlinear and Multiband Analysis via DWPT	Nonlinear and Statistic Parameters	F-score	SVM	C vs. MCI—79%
					C vs. ADM—81%
					C vs. ADA—84%
					MCI vs. ADM—89%
					MCI vs. ADA—94%
					ADM vs. ADA—78%
					All vs. All—57%

## Data Availability

Not applicable.
